# Focusing on scRNA-seq-Derived T Cell-Associated Genes to Identify Prognostic Signature and Immune Microenvironment Status in Low-Grade Glioma

**DOI:** 10.1155/2023/3648946

**Published:** 2023-05-31

**Authors:** Jiayu Wen, Qiaoyi Huang, Jiuxiu Yao, Wei Wei, Zehui Li, Huiqin Zhang, Surui Chang, Hui Pei, Yu Cao, Hao Li

**Affiliations:** ^1^Wangjing Hospital of China Academy of Chinese Medical Sciences, Beijing 100020, China; ^2^Graduate College, Beijing University of Chinese Medicine, Beijing 100020, China; ^3^Department of Geriatrics, Xiyuan Hospital, China Academy of Chinese Medical Sciences, Beijing 100089, China; ^4^First Clinical College, Shandong University of Traditional Chinese Medicine, Jinan 250011, China

## Abstract

**Background:**

The clinical outcomes of low-grade glioma (LGG) are associated with T cell infiltration, but the specific contribution of heterogeneous T cell types remains unclear.

**Method:**

To study the different functions of T cells in LGG, we mapped the single-cell RNA sequencing results of 10 LGG samples to obtain T cell marker genes. In addition, bulk RNA data of 975 LGG samples were collected for model construction. Algorithms such as TIMER, CIBERSORT, QUANTISEQ, MCPCOUTER, XCELL, and EPIC were used to depict the tumor microenvironment landscape. Subsequently, three immunotherapy cohorts, PRJEB23709, GSE78820, and IMvigor210, were used to explore the efficacy of immunotherapy.

**Results:**

The Human Primary Cell Atlas was used as a reference dataset to identify each cell cluster; a total of 15 cell clusters were defined and cells in cluster 12 were defined as T cells. According to the distribution of T cell subsets (CD4+ T cell, CD8+ T cell, Naïve T cell, and Treg cell), we selected the differentially expressed genes. Among the CD4+ T cell subsets, we screened 3 T cell-related genes, and the rest were 28, 4, and 13, respectively. Subsequently, according to the T cell marker genes, we screened six genes for constructing the model, namely, RTN1, HERPUD1, MX1, SEC61G, HOPX, and CHI3L1. The ROC curve showed that the predictive ability of the prognostic model for 1, 3, and 5 years was 0.881, 0.817, and 0.749 in the TCGA cohort, respectively. In addition, we found that risk scores were positively correlated with immune infiltration and immune checkpoints. To this end, we obtained three immunotherapy cohorts to verify their predictive ability of immunotherapy effects and found that high-risk patients had better clinical effects of immunotherapy.

**Conclusion:**

This single-cell RNA sequencing combined with bulk RNA sequencing may elucidate the composition of the tumor microenvironment and pave the way for the treatment of low-grade gliomas.

## 1. Introduction

In the brain and other parts of the central nervous system, gliomas are the most common primary malignant tumors [1]. According to the World Health Organization, gliomas were mainly classified into four levels and the higher grade notified the poor prognosis [2]. According to routine histopathology, low-grade gliomas are less malignant, usually in WHO grade 2 and 3 patients [3]. The characteristics of low-grade gliomas were their highly invasive nature, their difficulty in surgical resection, their recurrence, and their rapid progression to malignancy [4]. Several biomarkers were widely used to define a subtype which was correlated to a great prognosis like IDH1 and IDH2 [5]. LGG with both mutation of IDH1 and IDH2 and deficiency of chromosome of arms 1p and 19q have better therapeutic effect to radiochemotherapy than other LGG without these mutations [6]. Although more and more LGG-like biomarkers have been widely explored and applied in clinical practice, common biomarkers are still unable to effectively delineate the heterogeneity of tumor microenvironment [7]. Immunotherapy still has limited clinical benefits in LGG patients. Therefore, it is important to find an effectively prognostic biomarker or therapy target for the therapy of LGG patients.

TME were composed of numerous cell types including cancer cells, bone marrow-derived inflammatory cells, lymphocytes, blood vessels, and the extracellular matrix which were made up of collagen and proteoglycans [8]. The components of TME play an important role in the progression and invasion of tumors [9]. The alterations of TME not only impact the development of tumor but also could become biomarkers for prognosis and immunotherapy [10]. T cells, a subtype of immune cells, play an important role in innate immune and adaptive immune systems [11]. In the progression of cancer, the interactions between TME and T cells have a great influence on the development of tumors [12]. Poor vascular differentiation and cancer cell metabolism in the TME, which contribute to hypoxia, accumulation of metabolic waste, and insufficient energy supply, lead to the anergy of effector T cells to recognize and kill cancer cells [13]. T cells are also one of the important targets for immunotherapy. Stromal cells of TME mediate the coexistence of T cells and cancer cells which results in the immune escape of cancer cells and reduces the effect of immunotherapy [14]. Therefore, the study for T cells in TME is of great significance for the future search of tumor therapy.

Several immune cell populations in the TME can now be revealed molecularly through single-cell RNA sequencing (scRNA-seq) technology [15]. Previous studies have shown that screening immune cell subsets for relevant molecular signals based on RNA-seq data can help predict clinical outcomes and implement personalized medicine [16]. The aim of this study is to predict the T cell marker genes, construct a prognostic model, and evaluate the immunotherapy effect in patients with LGG.

## 2. Method

### 2.1. Data Collection

A total of 983 samples were enrolled in our investigation. Ten LGG tissues with scRNA-seq data were obtained from GSE138794 in GEO database, which were used to identify the T cell markers of LGG. The Cancer Genome Atlas (TCGA) transcriptome matrix (FPKM format) and clinical information of 481 LGG samples were obtained from the TCGA-LGG cohort to construct prognostic signatures. In addition, CGGA693 and CGGA325 cohorts were collected from the Chinese Glioma Genome Atlas (CGGA) database. The cohorts contained 332 and 162 patients, respectively, which were used as external validation cohorts to verify the prognostic model. In addition, GSE16011 was also included in this research to verify the accuracy of the model. As in our previous study, the microarray data was processed [17]. To make comparisons between samples easier, TCGA RNA sequencing data were converted to transcripts per kilobasemillion (TPM) values. To eliminate differences between batches, we used the “sva” package in R software for normalization. To ensure the availability and reliability of the data, strict inclusion and exclusion criteria were established for this study. Inclusion criteria were as follows: (1) the pathological results showed glioma, (2) complete genomic expression level data were included, and (3) clear reporting of pathological conditions and follow-up. Exclusion criteria were as follows: (1) other pathological types and (2) concurrent primary tumors from other sites. In addition, three immunotherapy cohorts (PRJEB23709, GSE78820, IMvigor210) were used to explore the immune treatment effect.

### 2.2. Identification of T Cell Marker Genes by scRNA-seq Analysis

scRNA-seq data were preprocessed, and three cells were excluded with less than 200 genes and gene expression only in individual cells. The different scRNA-seq datasets were corrected by the Harmony algorithm. The FindNeighbors function is used to distinguish cell subsets. The T-SNE function is used to show the distribution of cell subsets, and the single R package is used to annotate cell subsets. T cell marker genes were determined by screening criteria of adjusted *p* < 0.05 and |log2*FC*| > 1.

### 2.3. Construction of the Prognostic Model of T Cell Marker Genes

The transcriptional profiles of T cell marker genes were obtained based on single-cell data. LASSO algorithm was used to reduce the correlation between T cell marker genes and play a role in defitting. Subsequently, the multivariate Cox regression analysis algorithm was used to assign the coefficient of each gene to construct the prognosis model of T cell marker genes, in which the TCGA cohort was used as the training group and the CGGA cohort was used as the validation group. The risk values of key genes in the prognostic model are presented by dendrogram.

### 2.4. Tumor Microenvironment Landscape

To observe the overall landscape of immune cells in different T cell subsets, we used a variety of machine learning algorithms, including TIMER, CIBERSORT, QUANTISEQ, MCPCOUTER, XCELL, and EPIC. These algorithms can predict the content of immune cells based on transcriptome expression levels and find regularities through simulation of different algorithms to explain that T cell-related genes' change in the proportion of immune cells in TME. Expression levels of immune regulators and HLA family genes in different T cell subsets were examined to calculate the correlation between RNAss, DNAss, and risk scores.

### 2.5. Evaluation of Immunotherapy Effect

Risk scores were assigned to each patient in the three immunotherapy cohorts mentioned earlier based on the formula of the model construction. Compare the risk scores of immunotherapy responders and nonresponders to determine whether the risk model can be used to evaluate the effect of immunotherapy. In addition, the bar graph shows the AUC values used to predict the expression of individual cell subsets or molecules.

### 2.6. Statistics

All data analysis was analyzed with R software. GSEA algorithm was used to calculate the abundance of immune cell infiltration. Student's *t*-test was used to compare the differences between the two groups, and all statistical data were normally distributed. The PCA algorithm was used to render individual distribution. *p* < 0.05 was considered significant.

## 3. Results

### 3.1. Identification of T Cell Marker Gene Expression Profiles

**Figure 1:**
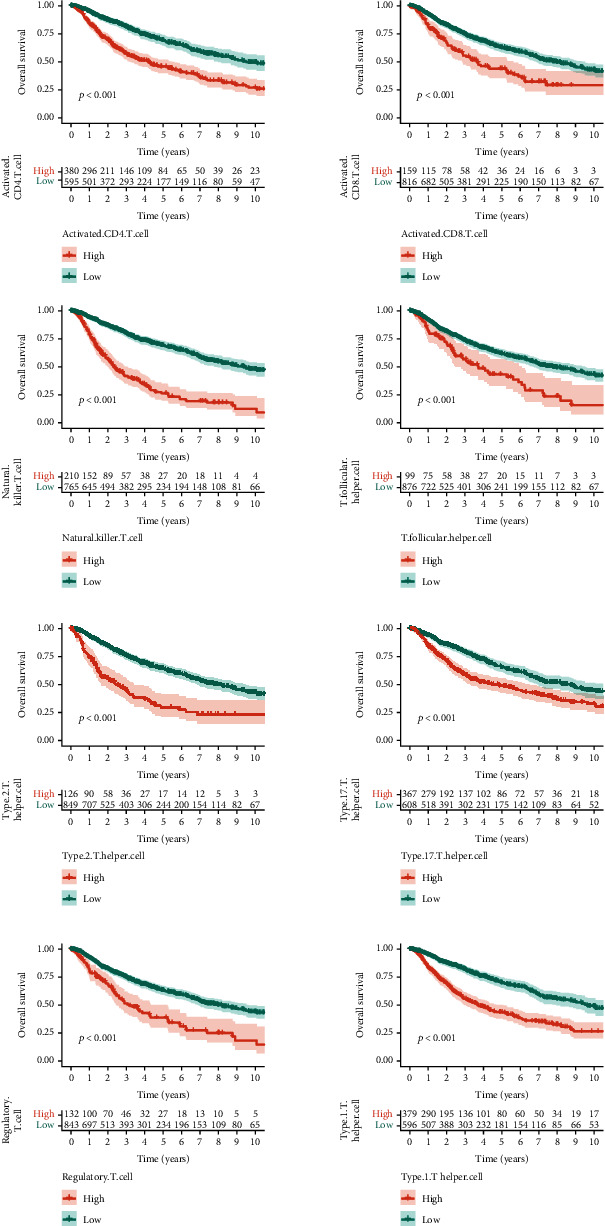
K-M survival analysis.

**Figure 2:**
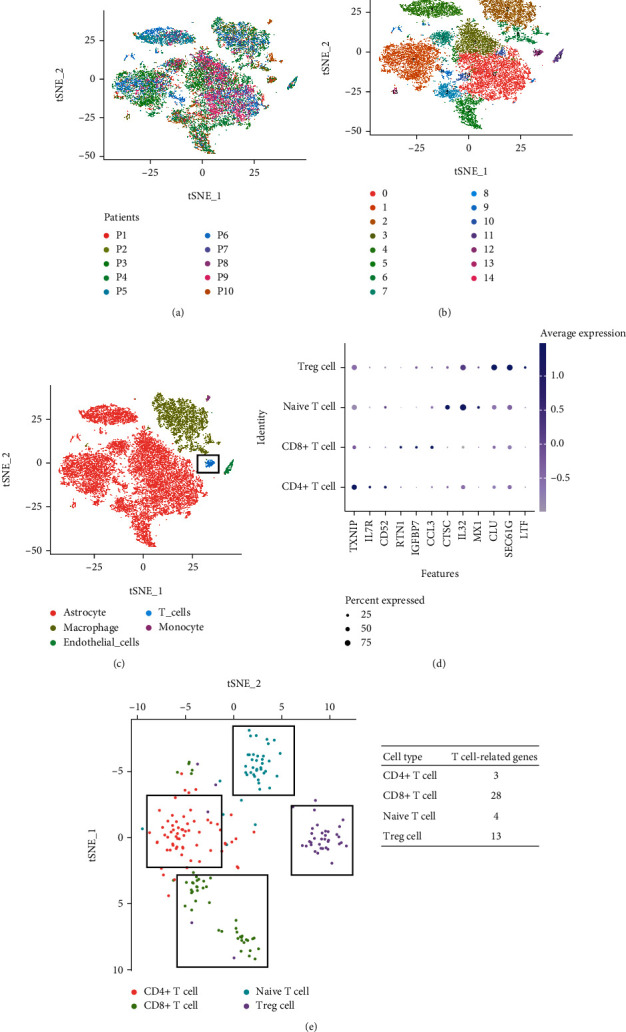
Identification of T cell marker gene. (a) The T-SNE showed the distribution of cell in ten patients. (b) 15 cell subsets were presented by T-SNE algorithm. (c) According to the expression level of marker genes, T-SNE algorithm drew 5 cell subsets. (d) T cell-related gene expression in four T cell subsets were plotted. (e) The T-SNE plot shows the cell distribution after reannotation of T cell subsets.

We calculated the immune cell infiltration score according to the ssGSEA algorithm and divided the patients into high and low immune cell infiltration groups according to the median immune cell infiltration score. The K-M results suggested that the infiltration level of T cell subsets had a great impact on the clinical outcome of patients (Figure 1). In addition, we calculated the content of immune cell subsets in different WHO grades and found that T cell subsets differed significantly in G2 and G3 grades. Cell distribution profiles of scRNA-seq data from GSE138794 are shown in Figure 2(a). To reduce the dimension, the top 1500 variable genes were selected and PCA was performed. A total of 15 cell clusters were identified, and cells in cluster 12 were defined as T cells by the Human Primary Cell Atlas (Figures 2(b) and 2(c)).  Figure 2(d) shows the expression of specific markers in various T cell subsets. TXNIP was mainly expressed in CD4+ T cells; CTSC and IL32 were mainly expressed in Naïve T cells; and CLU and SEC61G were mainly expressed in Treg T cells. In the CD4+ T cell subtype, there were 3 genes associated with T cell, and the remaining subsets were 28, 4, and 13, respectively, which were defined as T cell marker genes for subsequent analysis.

### 3.2. Prognostic Model

**Figure 3:**
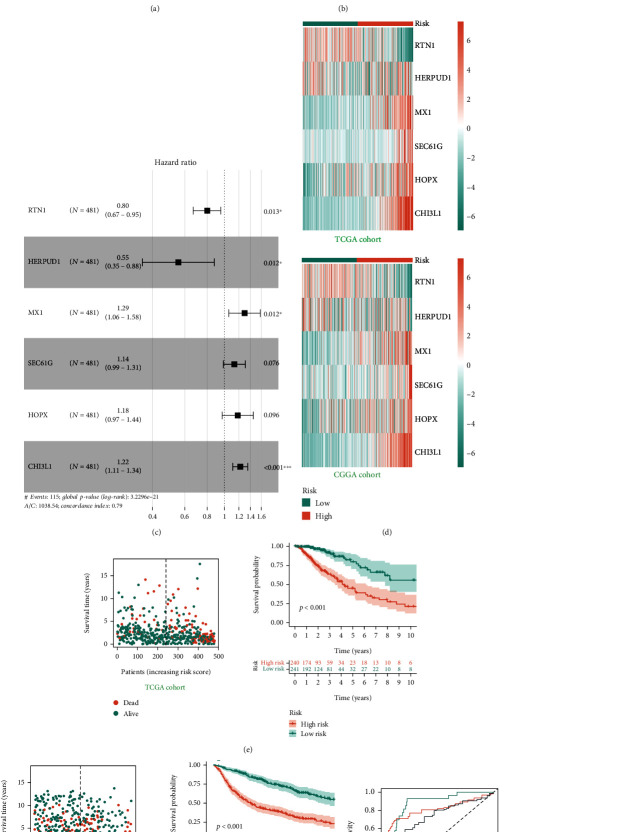
T cell-related model construction. (a) The PCA showed the distribution of patients in each cohort. (b) The LASSO algorithm for screening candidate genes. (c) The tree diagram shows the genes used to construct the prognostic model and their hazard values. (d) Heatmaps show the expression of prognostic genes in different risk groups. (e) Risk score and prognostic status of patients in the TCGA cohort. (f) Risk score and prognostic status of patients in the CGGA cohort. (g) The ROC curve for this model in the TCGA cohort. (h) The ROC curve for this model in the CGGA cohort.

PCA results showed that the three cohorts had batch effects distributed in different regions. As shown in Figure 3(a), after the batch effect was removed, data of three cohorts were at a consistent level. After the LASSO regression analysis, 10 genes were finally obtained. A multivariate Cox regression analysis screened candidate genes for model construction and calculated coefficient (Figure 3(b)). According to the expression values of candidate genes and corresponding coefficient, the model formula was constructed as follows:

(1) $$\begin{array}{c} \text{Riskscore}=0.257*\text{MX}1+0.127*\text{SEC}61\text{G}+0.168*\text{HOPX}+0.199*\text{CHI}3\text{L}1-0.222*\text{RTN}1-0.590*\text{HERPUD}1. \end{array}$$

The tree map shows the risk values of candidate genes, in which RTN1 and HERPUD1 are protective factors and MX1, SEC61G, HOPX, and CHI3L1 are risk factors (Figure 3(c)). The heatmap shows the expression levels of candidate genes between the high- and low-risk groups. RTN1 is highly expressed in the low-risk group, but MX1, SEC61G, HOPX, and CHI3L1 are highly expressed in the high-risk group. The expression trend of candidate genes in TCGA and CGGA cohorts is consistent in Figure 3(d). The dot plot shows the distribution of risk scores and clinical outcomes for each patient. With increase of the risk score, the mortality rate increases.

The K-M survival curve shows that patients in the high-risk group has a shorter survival time than those in the low-risk group (Figures 3(e) and 3(f)). The ROC curve shows that the prediction ability of the prognostic model at 1, 3, and 5 years in the TCGA cohort is 0.881, 0.817, and 0.749, respectively, and the prediction ability at 1, 3, and 5 years in the CGGA cohort is 0.749, 0.751, and 0.734, respectively (Figures 3(g) and 3(h)). In addition, we selected GSE16011 to further verify our prognostic model, and the results showed that it was consistent with the above, with the predictive power of up to 0.903, 0.818, and 0.776 at 1, 3, and 5 years, respectively (Figure S1).

**Figure 4:**
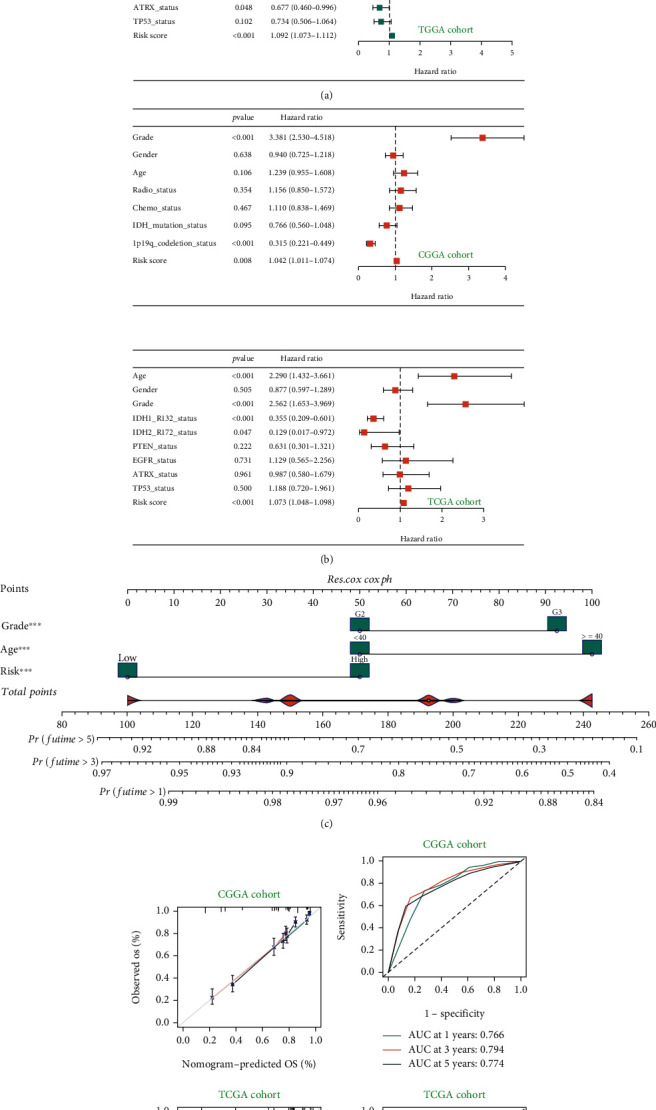
Nomogram model construction and verification. (a) The univariate regression analysis verified the predictive performance of the prognostic model. (b) The multivariate regression analysis verified the predictive performance of the prognostic model. (c) Construction of the nomogram model, including grade, age, and risk score. (d) The decision curve analysis was used to evaluate the predictive performance of the nomogram model. (e) The ROC curve was used to evaluate the predictive ability of the nomogram model for 1-, 3-, and 5-year survival prognosis.

Univariate and multivariate Cox regression analysis showed that the risk score model was an independent prognostic factor (Figures 4(a) and 4(b)). Multiple factors could be used to predict clinical outcomes. A nomogram model was constructed with risk score, grade, and AGE to predict clinical outcomes of patients (Figure 4(c)).  Figure 4(d) shows the relationship between the expected results and the actual observed values. The angle close to 45% represents a high accuracy. The ROC curve shows that the prediction ability of the prognostic model at 1, 3, and 5 years in the TCGA cohort is 0.829, 0.828, and 0.800, respectively, and the prediction ability at 1, 3, and 5 years in the CGGA cohort is 0.766, 0.794, and 0.774, respectively. The prediction performance was significantly improved (Figure 4(e)).

### 3.3. Correlation between Prognostic Models and TME

**Figure 5:**
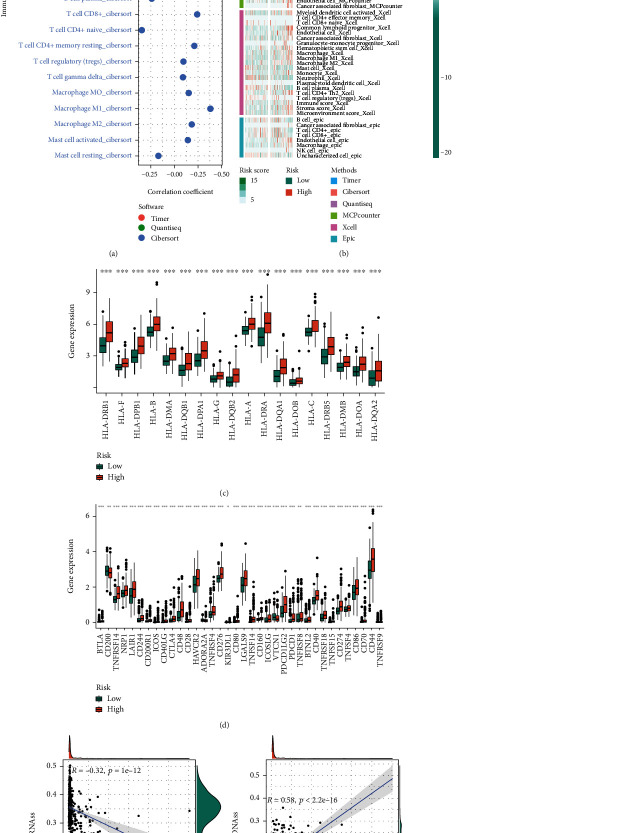
Tumor microenvironment assessment. (a) Correlation between risk score and immune cell infiltration. Algorithms: TIMER, QUANTISEQ, and CIBERSORT. (b) Heatmap shows the infiltration of immune cells in the high- and low-risk groups. Algorithms: TIMER, CIBERSORT, QUANTISEQ, MCPCOUNTER, XCELL, and EPIC. (c) The expression level of HLA gene family in the high- and low-risk groups. (d) Expression level of immunomodulator in the high- and low-risk groups. (e, f) The correlation between risk score and RNAss and DNAss scores.

As shown in Figure 5(a), the risk score is positively correlated with effector cells such as B cell and T cell, as well as M2 macrophages, but it is difficult to judge whether risk score exerted antitumor immunity or inhibited tumor immunity. The heatmap shows higher levels of immune cell infiltration in the high-risk group than those in the low-risk groups (Figure 5(b)). The expression of immunomodulators such as CD276, CTLA4, and HLA family molecules was higher in the high-risk group than that in the low-risk group (Figures 5(c) and 5(d)). The stemness index scores of RNAss were obtained based on transcriptome expression data, while those of DNAss were obtained based on methylation data. The risk score was negatively correlated with RNAss and positively correlated with DNAss (Figures 5(e) and 5(f)).

### 3.4. Immunotherapy Performance in Prognostic Models

**Figure 6:**
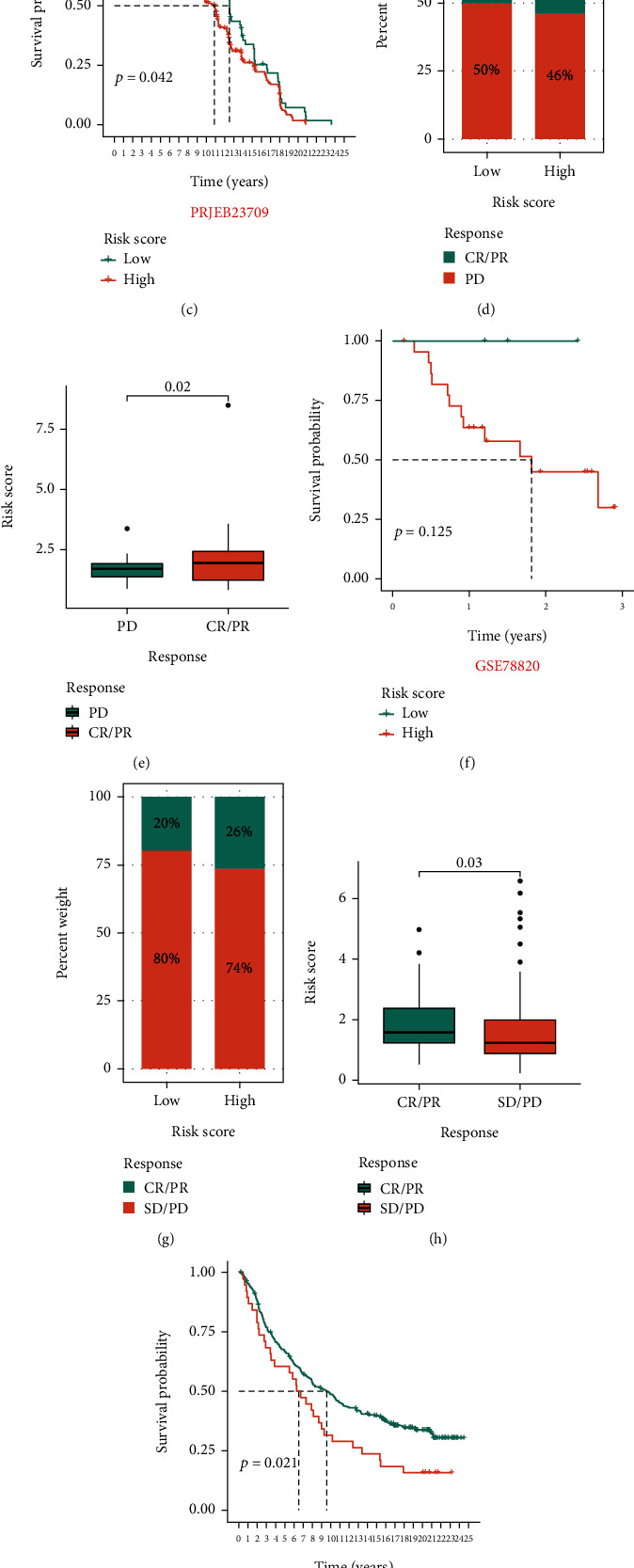
Evaluation of immunotherapy effect. (a–c) In the PRJEB23709 cohort, the bar chart shows the proportion of patients in the high- and low-risk groups who responded to immunotherapy. The box plot shows the risk score for different immunotherapy effects. The K-M survival analysis shows the clinical outcome of the high- and low-risk groups. (d–i) In the GSE78820 and IMvigor-210 cohorts. The bar chart shows the proportion of patients in the high- and low-risk groups who responded to immunotherapy. The box plot shows the risk score for different immunotherapy effects. The K-M survival analysis shows the clinical outcome of the high- and low-risk groups. (j) The bar chart shows the AUC values for each biomarker used to predict immunotherapy.

In the three immunotherapy cohorts of PRJEB23709, GSE78820, and IMvigor210, it was found that high-risk patients had better clinical effects on immunotherapy, and the median risk value of patients who responded to immunotherapy was higher than that of patients who did not respond to immunotherapy. Moreover, K-M curves showed that the overall survival time of high-risk patients was shorter than that of low-risk patients (Figures 6(a)–6(i)).  Figure 6(j) shows that the prediction performance of our prognostic model for immunotherapy response was 0.67 (Custom Geneset), which was lower than that of the immunodetection point (CD274) but higher than that of other T cells.

## 4. Discussion

scRNA-seq can precisely and rapidly determine the gene expression patterns of tens of thousands of individual cells [18]. Traditional bulk RNA-seq technology can only reflect the average expression level of genes in the population cells, which is difficult to mask the expression heterogeneity among different cells [19]. With scRNA-seq technology, all genes in a genome can be examined at the single-cell level, which is very helpful for studying cell expression heterogeneity [20]. In this study, scRNA-seq was used to process and analyze the glioma data in the public database, and the role of T cell marker gene in LGG was deeply explored. Based on the selected T cell marker genes, we constructed a prognostic model to predict clinical outcome and immunotherapy effect, and its prediction performance in 1, 3, and 5 years was 0.881, 0.817, and 0.749, respectively. In general, for healthy tissues and organs, the higher the degree of immune cell infiltration, the better the effect of antitumor and targeted killing [21]. The brain has a blood-brain barrier, which makes it difficult for immune cells to enter the brain [22]. Therefore, only low-grade gliomas have the good prognosis, but high-grade gliomas may destroy the blood-brain barrier and infiltrate more immune cells, directly leading to the poor prognosis of high-grade gliomas. Meanwhile, in low-grade gliomas, a high degree of immune cell infiltration is associated with poor clinical outcomes. This study found that LGG patients with high-risk scores had a higher degree of immune cell infiltration, and patients with high-risk scores had poorer clinical outcomes. T cell marker genes may serve as biomarkers to predict disease progression.

Another popular approach in immunotherapy is immune checkpoint blockade (ICB), which makes unprecedented advance in cancer treatment [23]. Interactions between ligands and receptors regulate ICBs in the immune system [24, 25]. In addition to regulating the duration and amplitude of physiological immune responses, it also maintains autoimmune tolerance. As a result, the immune system will not damage and destroy normal tissue [26, 27]. With the advent of immune checkpoint inhibitors (ICIs), mainly antiprogrammed cell death protein 1/programmed cell death ligand 1 (PD-1/PD-L1) and anticytotoxic T-lymphocyte-associated antigen-4 (CTLA-4) monoclonal antibodies have made great progress in the field of research related to certain types of cancer [28]. Both activated cytotoxic T lymphocytes to enhance antitumor response [29]. There is increasing evidence that molecule inhibitors that target carcinogenesis play a role far beyond the biological behavior of tumors [30, 31]. Our study found that the expression levels of immune modulators (such as PD-1 and PD-L1) and HLA family in the high-risk group were higher than those in the low-risk group. Based on three immunotherapy cohorts, the proportion of patients in the high-risk group who responded to immunotherapy was higher than that in the low-risk group. It is conceivable that a combination of T cell-based marker gene inhibitors and immune checkpoint inhibitors may benefit patients with LGG. In addition, bioinformatics methods were used to analyze the expression and prognosis of T cell marker genes in glioma.

In our investigation, we first performed a comprehensive study of scRNA-seq of patients with LGG to identify prognostic signatures and immune environment status. We identified T cell marker gene signature (TCMGS) to establish a survival model to evaluate the progression of LGG. Besides, based on the expression of TCMGS, we validated our prognostic model in an independent cohort which was downloaded from the Gene Expression Omnibus (GEO) database. Our study identifies that TCMGS may become the new target for the prognosis and treatment of LGG progression in the future.

There are some limitations in the study. First of all, our research is based on the mining of existing public databases with artificial bias. Secondly, possible pathogenic pathways are only proposed in this study, which requires further experimental verification. Finally, animal experiments are needed to test the hypothesis of drug combination before applying it to patients. Future studies of the relationship between T cell marker genes and cancer development and progression may focus on more discoveries of significant prognostic and even therapeutic value.

## Data Availability

Data were obtained from CGGA, TCGA, and GEO, as detailed in the methodology section.

## Supplementary Materials

Supplementary Materials Figure S1: external cohort validation (GSE16011). (A) The K-M survival analysis showed the clinical outcome of the high- and low-risk groups. (B) The ROC curve was used to evaluate the predictive ability of the nomogram model for the 1-, 3-, and 5-year survival prognosis.
