# Supplementary figures and images for: Focusing on scRNA-seq-Derived T Cell-Associated Genes to Identify Prognostic Signature and Immune Microenvironment Status in Low-Grade Glioma
Source: Mediators Inflamm. 2023 May 31;2023:3648946. doi: 10.1155/2023/3648946 (PMC10247320; doi:10.1155/2023/3648946)

**A**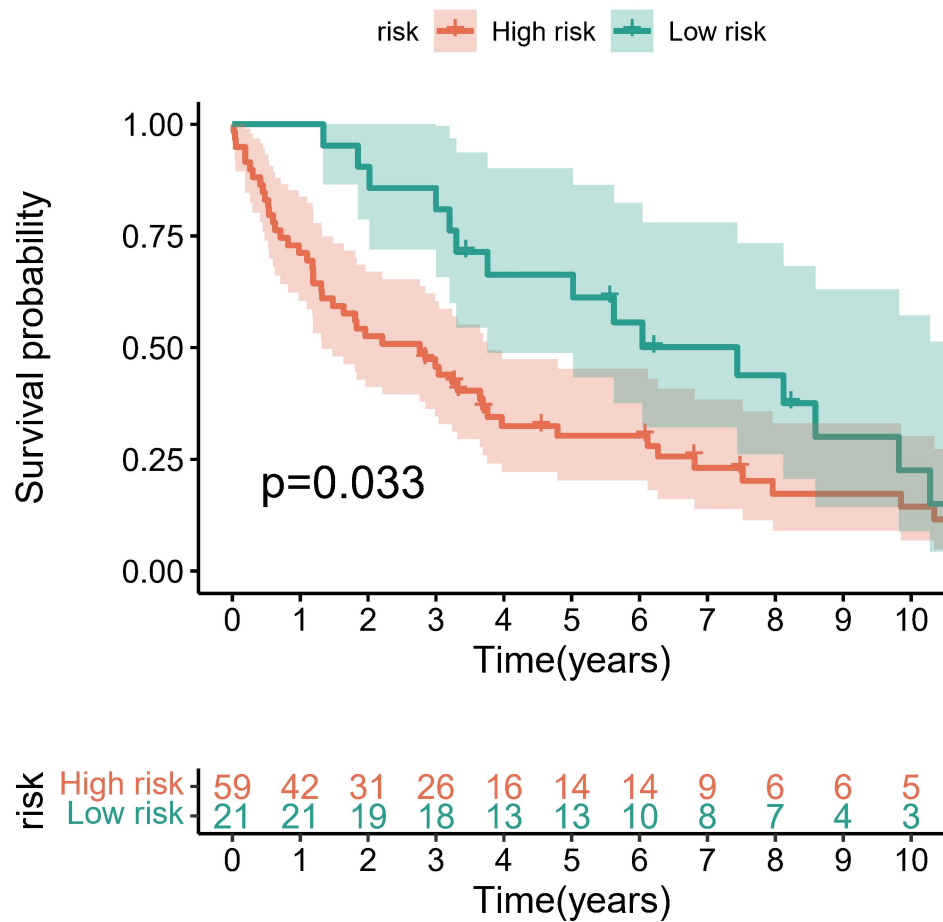**B**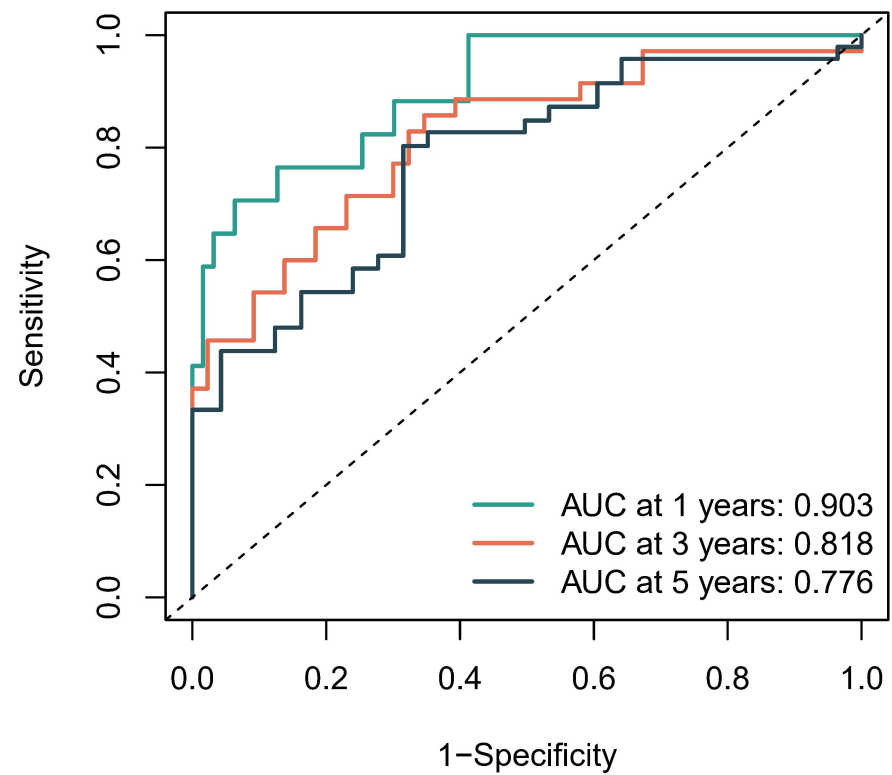

Supplement: Supplementary Materials — Figure S1: external cohort validation (GSE16011). (A) The K-M survival analysis showed the clinical outcome of the high- and low-risk groups. (B) The ROC curve was used to evaluate the predictive ability of the nomogram model for the 1-, 3-, and 5-year survival prognosis. [file 3648946.f1.pdf]
